# A Low-Cost Educational Intervention for Rural Hypertension Control: Lessons for National Scale-Up

**DOI:** 10.7759/cureus.85239

**Published:** 2025-06-02

**Authors:** Anubhav Mondal, Richa Kapoor

**Affiliations:** 1 Community Medicine, Vardhman Mahavir Medical College and Safdarjung Hospital, New Delhi, IND

**Keywords:** blood pressure control, education, follow-up, hypertension, intervention

## Abstract

Hypertension poses a major public health challenge in rural India, exacerbated by limited awareness and healthcare access. This quasi-experimental study among 110 hypertensive patients at a rural health center in Delhi assessed the impact of a low-cost, multifaceted educational intervention comprising flipchart-based counselling, voice calls, and Short Message Service (SMS) reminders. The intervention significantly improved blood pressure control (from 14.7% to 52.9%), medication adherence, physical activity, and dietary practices. Improvements were statistically significant, and participants with normal or reduced body mass index (BMI) showed better outcomes. The approach was simple and scalable and aligned with the National Programme for Prevention and Control of Non-Communicable Diseases goals. Persistent gaps in routine screenings and lifestyle adherence suggest a need for qualitative research and enhanced follow-up mechanisms.

## Editorial

Hypertension continues to pose a significant public health challenge in India, particularly in rural settings where healthcare access and awareness are limited [1]. Recent advances showcasing the promise of community-based strategies, including mHealth interventions, in addressing non-communicable diseases have drawn considerable attention. In this context, we wish to share insights from our quasi-experimental study involving 110 hypertensive patients at the Rural Health Training Centre in Najafgarh, Delhi. Our study evaluated the impact of a novel, low-cost, multifaceted educational intervention on blood pressure control, self-care practices, and treatment adherence in a resource-constrained rural population.

The study employed a multifaceted educational intervention to comprehensively educate hypertensive patients on self-care practices aimed at lifestyle modification and improved treatment adherence for blood pressure control. The intervention comprised three components: flipchart-based counselling, voice messaging, and text messaging.

In the flipchart component, participants received one-on-one counselling for 20 minutes with the aid of a validated flipchart. The content included an overview of hypertension: what high blood pressure is and its common symptoms. Risk factors such as obesity, heredity, stress, sedentary lifestyle, smoking, excessive salt intake, and age were discussed in detail. The counselling also focused on control measures, particularly lifestyle modifications. Participants were informed about dietary modifications aligned with the Dietary Approaches to Stop Hypertension (DASH) recommendations. They were also encouraged to engage in regular physical activity such as brisk walking. In addition, emphasis was placed on medication adherence, with strategies suggested for maintaining a consistent medication schedule. The counselling concluded with a discussion of the possible complications of uncontrolled hypertension.

As part of the voice messaging component, participants received weekly voice calls reminding them to engage in key self-care practices, including a healthy diet, regular physical activity, reduced salt intake, and stress management. These messages served as regular reinforcements to support behavioral change.

Text messaging involved sending weekly reminders to participants, encouraging the timely intake of prescribed medications. These messages were concise and aimed at fostering routine adherence and accountability among the participants.

The program was designed to be easily deliverable by community healthcare workers, aligning with the objectives of the National Programme for Prevention and Control of Non-Communicable Diseases (NP-NCD) [2]. Our approach was rooted in the need for accessible, culturally appropriate education and consistent reinforcement to promote behavior change among individuals managing hypertension.

The mean age of the study population was 54.5 years, with a predominant representation of women (65.5%) and homemakers (50.9%). Comorbid conditions such as obesity (87.3%) and diabetes (50.4%) were highly prevalent, and only 14.7% had controlled blood pressure at the start of the study. Self-care practices were suboptimal: just 11.8% adhered to a low-salt diet, 24.5% engaged in physical activity, and 46.1% reported adherence to prescribed medications. Alarmingly, 97.3% and 91.8% of participants were not undergoing regular blood tests and eye checkups, respectively, despite being at risk for complications.

Despite these concerning baselines, the six-month follow-up showed statistically significant improvements across all primary outcomes. The mean systolic blood pressure reduced from 151.1 mmHg to 138.3 mmHg (P<0.001), and diastolic pressure dropped from 87.5 mmHg to 82.8 mmHg (P<0.001). The proportion of participants achieving controlled blood pressure increased to 52.9% from 14.7% at the baseline, demonstrating the effectiveness of the intervention.

Significant improvements were also observed in lifestyle behaviors and treatment adherence. Medication adherence rose from 46.1% to 70.5% (P=0.002), salt consumption decreased (P=0.002), and adherence to physical activity nearly doubled (P=0.002). Participants reporting good weight management practices also increased significantly from 19.6% to 45.1% (P<0.001). The intervention also successfully reduced alcohol consumption (P<0.001).

We believe this intervention stands out due to its practical strengths. First, it used inexpensive and scalable tools, that is, flipcharts, Short Message Service (SMS), and voice calls, making it adaptable for broader primary healthcare settings. The standardized use of validated instruments added methodological rigor. Moreover, the statistical analyses, including repeated-measures analysis of variance (ANOVA) and Cochran's Q test with post-hoc analysis, allowed for robust conclusions regarding longitudinal changes.

An important insight from our study was the role of body mass index (BMI) in blood pressure control. Participants with normal BMI were significantly more likely to achieve blood pressure control than those who were overweight or obese (P=0.008). Those who achieved BMI reduction during the intervention were also more likely to report controlled blood pressure (P=0.02). Multivariate logistic regression further confirmed these associations: participants with normal BMI were 5.12 times more likely (CI: 1.33-19.61) to control their blood pressure, while those with BMI reduction were 2.51 times more likely (CI: 1.09-5.77) to do so. These findings suggest that weight management interventions should be an integral part of hypertension control strategies and should be explicitly incorporated into NP-NCD guidelines.

While the intervention was effective, our study also identified important areas for improvement. Despite improvements in self-care practices, a significant proportion of participants still remained non-adherent to lifestyle modifications and medications. Furthermore, the majority of participants did not undergo regular blood tests (like liver function test, kidney function test, etc.) or eye checkups, which are critical components of hypertension management, especially for detecting end-organ damage. These gaps suggest the need for complementary strategies, such as improved and stronger referral systems and incentivized health screening programs.

Our study does have limitations. Time constraints prevented an in-depth exploration of the qualitative aspects underlying poor adherence and lifestyle non-compliance. Understanding the psychosocial barriers, cultural beliefs, economic constraints, and perceptions of chronic disease could yield valuable insights. Therefore, we strongly recommend further qualitative research to examine why a significant proportion of participants remained non-adherent despite the intervention. Future studies could use in-depth interviews or focus group discussions with both patients and healthcare providers to address these gaps. Despite observable improvements in self-care practices and blood pressure control, it is important to acknowledge that these positive outcomes may not be solely attributable to the educational intervention, considering this was a before-after quasi-experimental study. External influences such as medication change, concurrent health campaigns, or seasonal variations in diet may have also contributed to the change. Future studies incorporating a control group would provide stronger evidence and more robust results.

In conclusion, our findings reinforce the utility of structured, culturally contextualized educational interventions for hypertension management in rural India. The combined use of flipcharts, SMS, and voice calls led to measurable improvements in blood pressure, medication adherence, and self-care behaviors. These results align with and support the goals of NP-NCD, advocating for simple yet effective Information, Education, and Communication (IEC)-based strategies within the primary healthcare framework. Given its feasibility and impact, we recommend the upscaling of this intervention model to other settings. However, further exploration of patient-specific barriers and sustained follow-up mechanisms is essential to ensure long-term outcomes.

We propose the following recommendations based on our findings: First, integrate IEC-driven interventions like flipcharts, voice calls, and SMS into the national program, particularly at the sub-center and primary health center (PHC) levels. Second, emphasize BMI management in hypertension care pathways and community education modules, supported by periodic anthropometric monitoring and behavior change communication. Third, limit the intervention period to four months with at least two structured follow-ups in resource-limited settings, given that the greatest reduction in blood pressure was observed during the initial follow-ups. Fourth, ensure routine screenings for blood pressure complications, including regular blood and eye tests, by enhancing community awareness and strengthening referral linkages. Fifth, undertake qualitative research to explore reasons for persistent non-adherence to medication and self-care behaviors post-intervention. Sixth, focus future efforts on alcohol and tobacco cessation as part of an integrated approach to lifestyle modification in hypertensive patients. Lastly, monitor long-term outcomes to evaluate sustainability, with scope for revising and improving strategies based on patient feedback and changing health system dynamics.

We believe these insights and recommendations are relevant for public health practitioners, policymakers, and primary care physicians engaged in non-communicable disease prevention and control. The urgent need to scale simple, cost-effective, and evidence-based solutions for chronic disease management cannot be overstated, especially in rural and underserved communities.
